# Deep Seated Density Anomalies Across the Iberia‐Africa Plate Boundary and Its Topographic Response

**DOI:** 10.1029/2019JB018445

**Published:** 2019-12-11

**Authors:** I. Jiménez‐Munt, M. Torne, M. Fernàndez, J. Vergés, A. Kumar, A. Carballo, D. García‐Castellanos

**Affiliations:** ^1^ Group of Dynamics of the Lithosphere (GDL), Institute of Earth Sciences Jaume Almera, ICTJA‐CSIC Barcelona Spain; ^2^ Department of Earth and Ocean Dynamics University of Barcelona Barcelona Spain

**Keywords:** thermophysical properties of the lithosphere, upper mantle composition, potential fields, petrological modeling, Gibraltar Arc and Atlas Mountains Betic‐Rif orogenic system

## Abstract

The modes in which the lithosphere deforms during continental collision and the mechanisms involved are not well understood. While continental subduction and mantle delamination are often invoked in tectonophysical studies, these processes are difficult to be confirmed in more complex tectonic regions such as the Gibraltar Arc. We study the present‐day density and compositional structure of the lithosphere along a transect running from South Iberia to North Africa crossing the western Gibraltar Arc. This region is located in the westernmost continental segment of the African‐Eurasian plates, characterized by a diffuse transpressive plate boundary. An integrated and self‐consistent geophysical‐petrological methodology is used to model the lithosphere structure variations and the thermophysical properties of the upper mantle. The crustal structure is mainly constrained by seismic experiments and geological data, whereas the composition of the lithospheric mantle is constrained by xenolith data. The results show large lateral variations in the topography of the lithosphere‐asthenosphere boundary. We distinguish different chemical lithospheric mantle domains that reproduce the main trends of the geophysical observables and the modeled *P* and *S* wave seismic velocities. A sublithospheric body colder than the surrounding mantle is needed beneath the Betics‐Rif to adjust the measured potential fields. We link this body to the Iberian slab localized just to the east of the profile and having some effect on the geoid and Bouguer anomalies. Local isostasy allows explaining most of the topography, but an elastic thickness higher than 10 km is needed to explain local misfits between the Atlas and the Rif Mountains.

## Key Points

- Lithosphere structure and upper mantle thermophysical properties in the Gibraltar Arc‐Atlas is obtained using an integrated geophysical‐petrological model
- A sublithospheric thermo‐compositional anomaly is found across the Iberia‐Africa plate boundary (Gibraltar)
- The thick lithosphere beneath the Betics and Rif tectonic domain, related to the W‐E slab, pulls down the topography about 1,500 m

## 1 Introduction

**Figure 1:**
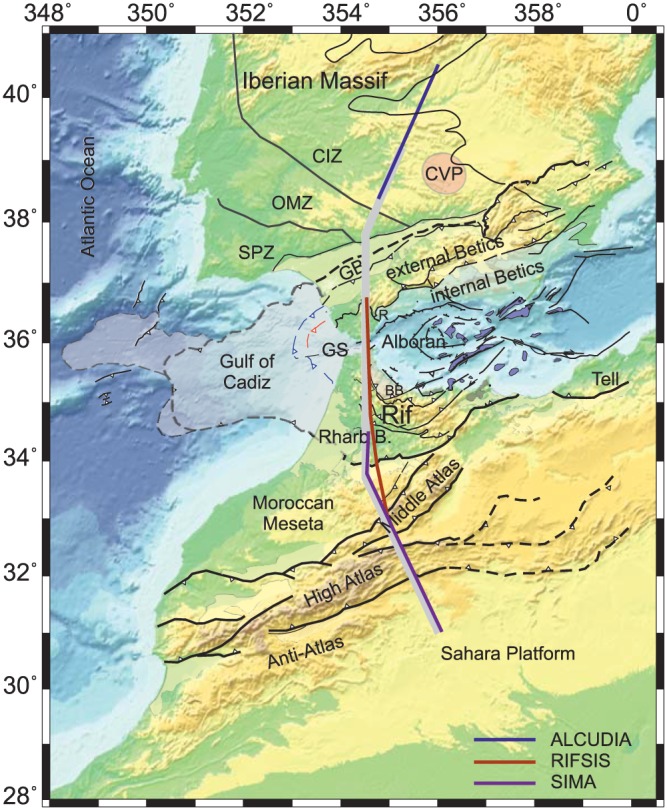
Elevation and main geological structures. CIZ, Central Iberian Zone; OMZ, Ossa Morena Zone; SPZ, South‐Portuguese Zone; CVP, Calatrava Volcanic Province; GB, Guadalquivir Basin; GS, Gibraltar Strait; BB, Beni Bousera; R: Ronda.

The target area of this study is located in the westernmost segment of the African‐Eurasia continental plate boundary, in the transition from the western Mediterranean to the Atlantic Ocean (Figure 1). The protracted convergence between the African and European plates accommodates over a widespread tectonically active deformation zone comprising the southern Iberian Massif, the Betic‐Rif arcuate orogen with its associated Alboran back‐arc basin and Guadalquivir and Rharb foreland basins, and the intracontinental Atlas Mountains. The entire region is affected by shallow, intermediate, and even deep seismicity showing normal, inverse, and strike‐slip focal mechanisms with hypocenter clustering related to major tectonic structures (e.g., Buforn et al., 2016; Custódio et al., 2016).

Seismic data and tomography studies show a complex structure characterized by large variations in crustal thickness (e.g., Diaz et al., 2016) and the presence of a partially broken lithospheric slab beneath the Betic‐Rif orogen (e.g., Bezada et al., 2013; Garcia‐Castellanos & Villasenor, 2011; Spakman & Wortel, 2004). Besides, there is a widespread volcanism over the study region affecting central Iberia and its eastern margin, the Alboran basin, and the Atlas Mountains (Figure 1). In addition to the Cenozoic volcanism in North Africa, the volcanic activity has concentrated during the mid and late Miocene and Pliocene to Recent showing orogenic and anorogenic affinities (e.g., Duggen et al., 2005; Lustrino et al., 2011; Lustrino & Wilson, 2007; Melchiorre et al., 2017).

There is growing consensus that the evolution of the region responds to a context of Cenozoic subduction of the Ligurian‐Tethys with further slab retreat and delamination, despite the fact that the initial subduction direction is still controversial as well as its areal extent and its current location in depth (e.g., Casciello et al., 2015; Chertova et al., 2014; Faccenna et al., 2004; van Hinsbergen et al., 2014; Vergés & Fernàndez, 2012).

In the last decade, the study area has been the subject of multiple geological and geophysical surveys including active and passive seismological experiments to unravel the structure of the crust and upper mantle and its geodynamic implications. Although results show consistent trends in the crustal thickness variations, there are conspicuous discrepancies between deep seismic sounding (DSS) profiles and receiver function (RF) analyses that locally can reach up to 8–10 km (e.g., Diaz et al., 2016). The depth of the lithosphere‐asthenosphere boundary (LAB) also shows noticeable discrepancies (some tens of kilometers) between Rayleigh wave tomography models (Palomeras et al., 2014, 2017), *P* and *S* RF analyses (de Lis Mancilla et al., 2015; Heit, 2017; Miller et al., 2013, 2015), and integrated geophysical models (Fullea et al., 2007, 2010; Jiménez‐Munt et al., 2011; Teixell et al., 2005; Torne et al., 2000, 2015; Zeyen et al., 2005).

The main aim of the study is to obtain, for the first time, a picture of the crust and upper mantle structure along a 945‐km‐long geotransect striking roughly N‐S from the South Iberian Massif to the Anti‐Atlas, crossing the Betic‐Rif orogen through the Gibraltar Strait, and the Atlas Mountains. The geotransect is aligned with recently acquired high‐resolution wide‐angle reflection and refraction seismic profiles complemented with the long‐standing deployment of broadband seismic stations (e.g., Ayarza et al., 2014; Bezada et al., 2014; Bonnin et al., 2014; Civiero et al., 2018; Ehsan et al., 2015; Gil et al., 2014; Palomeras et al., 2014; Thurner et al., 2014; Villaseñor et al., 2015).

To this end, we use the LitMod‐2D finite element code (Afonso et al., 2008) that, based on an integrated geophysical‐petrological methodology, allows determining the density, thermal, and seismic structure of the upper mantle down to 410‐km depth. Furthermore, we improved the code by allowing the incorporation of the anomalous sublithospheric bodies imaged by tomography along the geotransect, thus bridging the gap between previous integrated thermal models and seismic tomography interpretations.

The modeled crust and upper mantle structures are constrained by available data on elevation, Bouguer anomaly, geoid height, surface heat flow, chemical composition from mantle xenoliths, and seismic data including seismic refraction and wide‐angle reflection, RFs, and *P* and *S* wave tomographic models. Specific objectives of the study are (1) to propose a more reliable structure of the crust and the lithosphere mantle incorporating recent seismic and petrophysical‐geochemical constraints; (2) to analyze the influence of lateral changes of the lithospheric mantle composition on the resulting LAB geometry; (3) to compare the thermally, seismologically and petrological‐geophysical derived LABs; (4) to study the effects of sublithospheric mantle thermal perturbations imaged by tomography models and correlate them with the dynamic topography; and (5) to improve the knowledge of the deep structure of the Iberia‐Africa plate boundary active during the last 200 Myr involving strike‐slip, transtensional and compressional tectonics.

## 2 Highlights of the Geology of the Region

For modeling and discussion purposes, we consider three main domains: the southern Iberian Massif, the Betic‐Rif orogenic system, and the Atlas domain including the Moroccan Meseta, the Atlas Mountains, and the Anti‐Atlas. In what follows we particularly focus on the tectonic units crossed by the modeled geotransect.

### 2.1 The Southern Iberian Massif

The northern segment of the profile crosses the southern part of the Iberian Massif, an almost preserved Late Paleozoic Variscan‐Alleghanian orogen, and constituted by the Central Iberian Zone (CIZ), the Ossa Morena Zone (OMZ), and the South Portuguese Zone (e.g., Martínez‐Catalán, 2011; Murphy et al., 2016; Pérez‐Estaún & Bea, 2004) (Figure 1).

The CIZ is composed of Neoproterozoic metamorphic rocks unconformably covered with Paleozoic metasedimentary rocks and lower Paleozoic metavolcanics. Large post‐Variscan late Carboniferous granitoids intruded the CIZ crust. The CIZ is separated from the OMZ by a suture produced during the closure of a small Early Paleozoic oceanic domain (Azor et al., 1994; Burg et al., 1981; Gómez‐Pugnaire et al., 2003; Simancas et al., 2001). Late Precambrian and Paleozoic metasediments compose the OMZ including large outcrops of Cambrian‐Ordovician metaigneous rocks, Lower Carboniferous deposits and magmatic intrusions. The boundary between the OMZ and the South‐Portuguese Zone (SPZ) is inferred as the suture of the Rheic Ocean during the Devonian period (e.g., Pérez‐Cáceres et al., 2015). While the CIZ and the OMZ display significant deformation, magmatism, and metamorphism, the SPZ shows typical characteristics of a frontal accretionary prism with abundant Upper Carboniferous flysch synorogenic deposits and thin‐skin tectonics. The central SPZ corresponds to the Iberian Pyrite belt, metasediments of Devonian age (e.g., Pérez‐Cáceres et al., 2015). The mantle xenoliths of the Calatrava Volcanic Province (CVP) in central Spain represent peridotite fragments from the concealed subcontinental lithospheric mantle incorporated into a rising volcanic melt erupting onto the surface since the late Miocene (8.7–6.4 Ma) and notably since the Pliocene.

### 2.2 The Betic‐Rif Orogenic System

The central segment of the profile crosses the Betic‐Rif orogenic system, which shows a significant continuity along the Gibraltar region. Several features are very characteristic of this orogenic system as its arcuate geometry (Betic‐Gibraltar‐Rif arc), the abundance of high‐pressure/low‐temperature (HP/LT) metamorphic slices in the Internal domains of both Betics and Rif, the large outcrops of Ronda and Beni Bousera upper mantle peridotite bodies, and the marine Alboran Neogene extensional basin in the interior part of the arcuate orogenic system.

The NW‐SE trend of the Variscan tectonic grain is overprinted by the Betic orogenic system, which displays a consistent ENE‐WSW direction inherited from the Betic margin orientation along the Ligurian‐Tethys margin. The Betics consists of a typical subduction‐related fold‐and‐thrust belt constituted by the Guadalquivir basin, the External Betics (Prebetic shallow marine deposits thrusted by Subbetic deeper marine marly deposits), the Flysch Units comprising deep marine turbidites, and the Internal Betics formed by stacked tectonic slices of HP/LT metamorphic Upper Paleozoic‐Triassic rocks. The core of the Betic orogen is, however, formed by the marine Alboran basin filled up by a thick Miocene to present sedimentary succession and abundant volcanic intrusions (Duggen et al., 2008; Gómez de la Peña et al., 2018; Melchiorre et al., 2017).

The Guadalquivir basin is a narrow ENE‐WSW trending flexural basin associated with the Western and Central Betics. The top of the basement dips 2–4° toward the SE in the most external part of the basin and is filled up with middle Miocene to present sedimentary successions reaching more than 2.6 km of thickness in its western depocenter (Garcia‐Castellanos et al., 2002; Iribarren et al., 2009). The External Betics formed the Jurassic‐Lower Cretaceous extensional SE Iberian margin along the Ligurian‐Tethys Ocean (García‐Hernández et al., 1980). Along the transect, the External Betics and the Flysch Units form imbricate thrust belts with a NNW directed transport swinging to the west in the Gibraltar Strait showing a large number of long folds, cut by subsidiary thrusts with a large component of Upper Triassic salt diapirism (Iribarren et al., 2007).

The Internal Betics are composed by a stack of tectonic slices, constituted by HP/LT metamorphic Upper Paleozoic‐Triassic rocks, differentiating the nonmetamorphic Malaguide units on top of the tectonic pile, the Alpujarride units (mostly composed of Triassic) in the middle, and the Nevado‐Filabride units (mostly of Paleozoic composition) showing maximum burial depths of about 65 km. However, along the geotransect there is no evidence, at surface or at depth, of the presence of these units.

The Rif fold belt is composed of a system of roughly west to SSW directed thrust imbricates involving from Internal metamorphic allochthonous units and upper mantle peridotite Beni Bousera massif in the inner part of the belt to the fold‐and‐thrust cover system in the External Rif that corresponds to the deformed Jurassic NW Moroccan paleomargin and the Rharb foreland basin. In the study transect, the Rif is interpreted as a folded and thrusted Mesozoic cover (External Rif) thrusted above the Rharb foreland basin from which only a very narrow fringe appears in the line of the transect. The basement beneath the Rharb basin and partially beneath the Rif, crops out in the Variscan Western Meseta; a Paleozoic massif characterized by intense WNW directed folding and thrusting. Greenschist to amphibolite facies show the metamorphic grade and extensive synorogenic to late orogenic magmatism intruded the deformed units (e.g., Michard et al., 2010).

### 2.3 The Atlas Mountains

The Atlas Mountains are classically interpreted as a failed transtensional rift arm that formed as a result of the Pangea breakup and evolved through Late Permian‐Middle Triassic and Early Jurassic during the opening of the Central Atlantic (e.g., Hosseinpour et al., 2016; Schettino & Turco, 2009; Vially et al., 1994). The complex array of branches like the NE‐SW Middle Atlas and the ENE‐WSW High Atlas is consistent with the widespread extension occurring along the northernmost section of NW African plate. The transect crosses the Central High Atlas that resulted from the tectonic inversion of a salt‐related rift basin in which salt walls and salt anticlines grew since the lower Jurassic (Saura et al., 2014). Tectonic inversion involved the basement structure raising the whole basin at high altitude (up to 4,000 m) but preserving its initial layout with a limited shortening tightening the salt diapirs primarily. In the synclinal zones, 4,000 m of sediments of the lower and middle Jurassic are still preserved and reconstructions based on thermal maturation data indicate that the total sedimentary pile reached up to 5‐km thickness including Jurassic and a thin Cretaceous sequence (Moragas et al., 2018).

The present configuration of the Atlas in Morocco is the consequence of multiple large‐scale lithospheric events that occurred after the onset of the northward convergence of Africa at 83.5 Ma (e.g., Macchiavelli et al., 2017). These include lithospheric mantle thinning under the Central High Atlas and the long history of volcanism and magmatism during the Jurassic‐Cretaceous extensional period and its continuation during later shortening (e.g., Ayarza et al., 2014; Frizon de Lamotte et al., 2009; Fullea et al., 2010; Jiménez‐Munt et al., 2011; Missenard et al., 2006; Teixell et al., 2005). Alkaline volcanism is present throughout the chain including the Middle Atlas and the Anti‐Atlas and thus intruding in a wider area than the Atlas. The Cenozoic dated magmatism ranges from 45 to 0.5 Ma (e.g., Lustrino & Wilson, 2007).

The Anti‐Atlas chain is a broad anticlinorium, trending ENE‐WSW, parallel to the Alpine High Atlas chain that has been rejuvenated during Alpine compression. The Precambrian ENE trending inliers of the Anti‐Atlas folding expose rocks of the Neoproterozoic Pan‐African belt and of its Paleozoic foreland reaching 10 km of prefolding thickness (Gasquet et al., 2008, and references therein) and are partly covered by thin Cretaceous‐Neogene deposits.

## 3 Integrated Geophysical and Petrological Modeling

### 3.1 Method and Model Parameters

LitMod‐2D code (Afonso et al., 2008) is a forward scheme that combines petrological and geophysical modeling of the crust and upper mantle down to 410‐km depth within an internally consistent thermodynamic‐geophysical framework. The modeling domain is divided into crust and mantle bodies each of it with predefined thermophysical properties (crustal bodies) and chemical composition (mantle bodies). To reduce uncertainties, the density and geometry of the crust are mainly constrained from available seismic experiments and geological data, while the composition and geometry of the lithospheric mantle are mainly constrained by available xenolith data and tomography models. The chemical composition of the lithospheric mantle is defined within the NCFMAS system (Na_2_O‐CaO‐FeO‐MgO‐Al_2_O_3_‐SiO_2_), from which stable mineral assemblages are determined by minimizing the Gibbs free energy (Connolly, 2005). The physical properties of each mineral and of the bulk mantle (density, thermal expansion coefficient, elastic parameters, and thermal conductivity) are calculated as function of temperature, pressure, composition, and phase changes.

The heat transport equation through the lithosphere is solved using the finite elements method in steady state with the following boundary conditions: 0 °C at the surface; 1320 °C at the LAB; and no heat flow across the lateral boundaries of the model. To avoid unrealistic discontinuities between the conductive thermal gradient of the lithospheric mantle and the adiabatic thermal gradient of the asthenosphere, we define a 40‐km‐thick thermal buffer beneath the LAB with a temperature of 1400 °C at its base. The temperature at the base of the model (410 km) is set to 1520 °C. The temperature gradient below the thermal buffer layer is restricted to 0.35 < d*T*/d*z* < 0.50 °C/km; otherwise, the temperature at 410‐km‐depth is modified accordingly (see Afonso et al., 2008, for further details). As in Carballo et al. (2015), we incorporate sublithospheric thermocompositional anomalous bodies, derived from tomography models, to account for detached and sunk portions of the lithospheric mantle and/or regions of the asthenosphere with anomalous temperature.

Once the physical properties are determined for each mantle composition, gravity, geoid, elevation, surface heat flow, and *P* and *S* seismic velocities are computed and compared with the observed values. Gravity and geoid are calculated by using simple algorithms to each element of the mesh. Elevation is calculated assuming local isostasy referenced to a mid‐ocean ridge column with the compensation level at 410‐km depth. Sublithospheric anomalies can be mechanically coupled or decoupled to the overlying lithosphere depending on whether the vertical stresses related to its buoyancy are totally transmitted to surface elevation or not (see Carballo, Fernàndez, Torne, et al., 2015). Consequently, decoupling anomalies has no influence on the calculated isostatic elevation but does effect on geoid, gravity, and mantle seismic velocities calculations. Short‐wavelength variations of elevation along the modeled transect suggest that topography can be supported to some degree by flexural rigidity of the lithosphere. We filter the elevation misfits by using an elastic‐thin‐plate flexural isostatic approach, assuming a constant elastic thickness and a load distribution resulting from the pressure variations at the compensation level (more details on Jiménez‐Munt et al., 2010), using the code *tAo* (Garcia‐Castellanos, 2007; code available at https://github.com/danigeos/tao-geo). Large elastic thickness smooths out laterally the elevation misfit due to pressure changes at the compensation level. A reasonable elastic thickness for our study area is 10 km (Garcia‐Castellanos et al., 2002), which reduces the misfit below the standard deviation in topography along most of the profile (Figure 7).

### 3.2 Surface Geophysical Data

**Figure 2:**
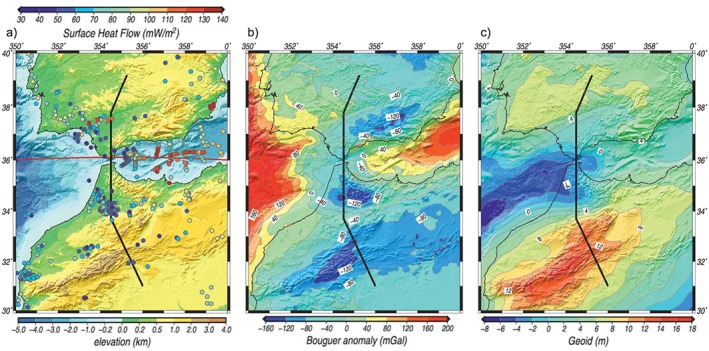
(a) Elevation and surface heat flow (dots), (b) Bouguer anomaly, and (c) geoid filtered at degree 10. Shadows represent the elevation. Gray line is the location of the transect from this work. Red line in (a) is the location of the W‐E profile shown in Figures 3b, 3d, and 3f.

Elevation data (Figure 2a) come from ETOPO1 (Amante & Eakins, 2009), a global elevation model of the Earth surface with 1 × 1‐min arc resolution available on the National Oceanic and Atmospheric Administration website (http://www.ngdc.noaa.gov/mgg/global/global.html). The three tectonic domains along the modeled transect show distinct elevation imprints. The northern sector of the profile that crosses the SE region of the stable Iberian Massif has an average altitude slightly above 500 m. The central sector runs along the west Betic‐Rif orogenic system and has an average elevation of 225 m with maximum and minimum values of 600 and −600 m in the External zones and Gibraltar Strait, respectively. Note that the transect does not cross the Internal Zones of the orogenic system where much higher elevations are observed (Figure 2a). In the southern sector, the Atlas intracontinental mountain belt is characterized by much higher topography with average elevation along the modeled transect of 2,000 m and maximum values of 2,700 m. From there topography smoothly descends toward the Anti‐Atlas Mountains where the average values are 700 m (Figure 2a).

The Bouguer gravity anomaly (Figure 2b) comes, from a recent compilation of gravity data in Iberia (Ayala et al., 2016) and from Hildenbrand et al. (1988) onshore Morocco. For the rest of Africa and offshore regions it is calculated applying the complete Bouguer correction to free air satellite data (Sandwell & Smith, 1997, updated 2007) using the software FA2BOUG (Fullea et al., 2008) with a density reduction of 2,670 kg/m^3^. Figure 2b shows that the southern region of the stable Iberian Massif is characterized by an anomalous gravity high (close to 80 mGal in the SW corner) that encompasses the Ossa‐Morena and South Portuguese zones, with the 0‐mGal contour roughly delineating the boundary between the CIZ and the Ossa‐Morena Zone (OMZ). The northern sector of the transect shows increasing values from −20 mGal in the CIZ to 10 mGal in the OMZ. The Gibraltar Arc region shows a very distinctive pattern delimited by an arcuate regional gravity low, with minimum values below −100 mGal in the Central Betics and the external Rif and eastern Rharb basin. To the south, an ENE‐WSW trending regional gravity low (up to −120 mGal) extends from the High Atlas to the east, covering the entire Algerian plateau. East and west of the Gibraltar Strait negative anomalies characterize the thick sedimentary sequences of the Alboran Basin and Gulf of Cadiz increasing rapidly to values exceeding 140 mGal toward the oceanic domains of the Algerian Basin and the Atlantic Ocean (Figures 1 and 2b).

Geoid height data come from ICGEM (Ince et al., 2019; http://icgem.gfz-potsdam.de) where we used the GECO model (Gilardoni et al., 2016) filtered up to degree and order 10, to retain geoid anomalies coming from lateral density variations within the crust and upper mantle to ~400‐km depth. Figure 2c shows that the stable Iberian Massif and the Atlas Mountains are characterized by relative highs (above 5 and 9 m, respectively), while the Gibraltar Arc domain shows positive values in its eastern sector and negatives to the west (<−1 m), reflecting the complexity of the deep structure of this region. Along the transect, the northern and southern sectors show two prominent geoid highs above 6 and 12 m, respectively, whereas its central sector is characterized by a geoid low of ~−4 m.

Heat flow data have been gathered from different surveys and compilations including seafloor heat flow measurements and water and oil exploration wells. The measured values show a high scatter mainly related to fluid flow and surface mass transport processes (Figure 2a). Along the northern section of the profile (Iberian Massif) there are not available heat flow measurements and therefore, we used estimations derived from Curie depth determinations, showing values of 65–70 mW/m^2^ (Andrés et al., 2018). In the central segment of the transect, heat flow decreases southward from 60–70 mW/m^2^ in the western Guadalquivir Basin to 40–50 mW/m^2^ in the External Betics (Fernàndez et al., 1998, b). In the Gibraltar Strait region, values range from 40–50 mW/m^2^ in the Gulf of Cadiz (Grevemeyer et al., 2009) to 55–65 mW/m^2^ in the westernmost Alboran Basin (Polyak et al., 1996). In Morocco inland, the average heat flow values range from 45–70 mW/m^2^ in the External Rif and Rharb Basin to 50–60 mW/m^2^ in the High Atlas (Rimi et al., 2005).

### 3.3 Moho Depth and Crustal Structure From Previous Studies

We have used the recently acquired wide‐angle reflection and refraction seismic profiles along the geotransect to constrain the geometry and seismic velocities within the crust and the uppermost mantle. In the Iberian Massif we use the ALCUDIA profile (Ehsan et al., 2015), whereas in the central segment we use the RIFSIS profile crossing the Rif and Middle Atlas (Gil et al., 2014). This profile partly overlaps the SIMA profile (Ayarza et al., 2014), which covers the Middle and High Atlas in the southern segment of the geotransect. The crustal structure along the RIFSIS profile was interpreted in combination with gravity data. In addition to these profiles, we projected onto the geotransect the updated compilation of Moho depth data covering the entire region derived from DSS profiles and RF analysis (Diaz et al., 2016).

To constrain the internal structure of the crust, we considered the ALCUDIA (Martínez Poyatos et al., 2012) and IBERSEIS (Simancas et al., 2003) seismic reflection profiles and related cross sections in the Iberian Massif, and the Marismas geological cross section through the Guadalquivir Basin and External Betics (Berástegui et al., 1998). The sedimentary thicknesses of Neogene basins of the Betics and Rif domains are based on Iribarren et al. (2007, 2009) and references therein.

Crustal density values come from previous studies (Gil et al., 2014) and using the velocity‐density envelopes defined by Brocher (2005). Thermal conductivities are taken from previous studies (e.g., Teixell et al., 2005; Torne et al., 2000, 2015; Zeyen et al., 2005), and radiogenic heat production comes from direct measurements in the Iberian Massif and Betics (Fernàndez, Beràstegui, et al., 1998) and from a global compilation of relevant crustal rocks (Vilà et al., 2010). Table 1 summarizes the assigned physical parameters to the crustal bodies.

**Table 1:** Physical Properties of the Different Tectonic Units Used in the Crustal Model Along the )

Tectonic units	*ρ* (kg/m3)	*K* (W/K·m)	*H* (μW/m3)
Atlas inverted Rift Basin	2,680	2.5	1.0
Rharb foreland Basin	2,600/2,300	2.6/2.4	1.0/0.85
Guadalquivir foreland basin	2,350	2.4	0.85
Upper Triassic Unit	2,300	2.5	1.1
Gibraltar Flysch units	2,500	2.6	1.0
Betic‐Rif thrust system	2,700	2.4	0.8
Granite Batholit	2,670	2.9	2.8
Upper Crust	2,750	2.7	1.74
Middle Crust	2,810	2.5	0.9
Lower Crust	2,950	2.1	0.2
Highly intruded lower crust	3,100	2.1	0.02

*Note*. Radiogenic heat production *H*; thermal conductivity *K*; and density *ρ*.

### 3.4 Mantle Characterization and LAB Depth From Previous Studies

**Figure 3:**
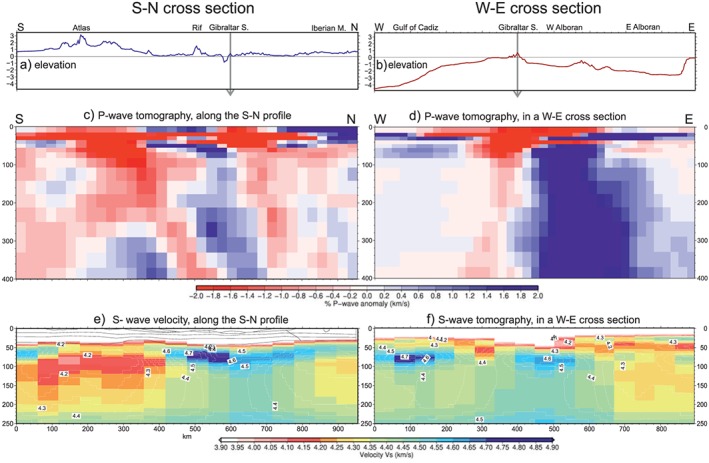
(a, b) elevation, gray arrows mark the crossing point between profiles; (c, d) *P* wave seismic tomography image from Bezada et al. (2013); (e, f) Absolute shear velocities from Palomeras et al. (2017). Along two cross sections, (a, c, and e) our N‐S profile (black line Figure 2a); and (b, d, and f) a W‐E profile crossing the Gibraltar Strait (red line Figure 2a).

The mantle characterization along the profile comes mainly from available global and regional tomography models, geochemical and isotopic analyses of xenolith samples, and Pn velocities derived from wide‐angle seismic profiles. *P* wave mantle velocities are derived from global and regional travel time and multiple‐frequency tomography models (e.g., Bezada et al., 2013; Bonnin et al., 2014; Civiero et al., 2018; Villaseñor et al., 2015). Figures 3c and 3d show *P* wave velocity anomalies obtained from Bezada et al. (2013) along the modeled profile and along a perpendicular W‐E profile crossing the Gibraltar Strait (location in Figure 2a). Positive velocity anomalies ~1% are located beneath the Betic‐Rif orogenic system imaging the subducting slab of the Gibraltar Arc region, clearly seen in the W‐E profile (Figure 3b). To the south, the Atlas Mountains region shows a prominent regional low velocity (−2%) from 40‐ to 120‐km depth.

Figures 3c and 3d show the shear velocity model along the geotransect and the perpendicular profile calculated from Rayleigh wave tomography using both ambient noise and teleseismic arrivals (Palomeras et al., 2017). As pointed out by these authors, high shear velocities (>4.5 km/s) beneath the Gibraltar Arc (Alboran domain, W‐Betics, and NW‐Rif) trace the subducting slab, which is surrounded by low upper mantle *Vs* values (<4.3 km/s) underneath the Iberian Massif and the Atlas region down to 200‐km depth. However, while low values (4.25 to 4.3 km/s) in the Iberian Massif extend from 70‐ to 200‐km depth, in the Atlas Mountains *Vs* values are <4.2 km/s covering a much wider and deeper region with Neogene volcanism, thus suggesting the presence of an anomalously thin lithosphere. Besides, we have also considered regional and global tomography models (e.g., Bonnin et al., 2014; Civiero et al., 2018; Villaseñor et al., 2015).

Further information on the geometry of the LAB come from previous 2‐D and 3‐D lithospheric models carried out in the study region using either a pure thermal approach (e.g., Fernàndez et al., 2004; Fullea et al., 2007; Jiménez‐Munt et al., 2011; Palomeras et al., 2011; Teixell et al., 2005; Torne et al., 2000, 2015; Zeyen et al., 2005) or a geophysical‐petrological approach (Carballo, Fernàndez, Torne, et al., 2015; Fullea et al., 2010).

Xenolith data along the modeled transect are restricted to the Calatrava Volcanic Province (CVP), located ~100 km east from the northern end of the transect, and the Middle Atlas. Geochemical analyses from the spinel lherzolite xenoliths in the CVP show a moderately fertile mantle with low degree of partial melting that underwent two metasomatizing events associated with subduction‐related melt and alkaline melt (Villaseca et al., 2010). The analyses of peridotite xenoliths recovered from Pliocene‐Quaternary alkaline basaltic melts in the Middle Atlas performed by Raffone et al. (2009) and El Messbahi et al. (2015) show an extreme lithological and chemical heterogeneity that is consistent with widespread and variable degree of metasomatism in their lithospheric mantle source.

## 4 Results

The forward modeling scheme consists of three main steps: (i) projection of all relevant data onto the geotransect; (ii) building up an initial model with crustal and mantle bodies and physical parameters; and (iii) modifying the initial model to reach the best fit with the observables.

The crustal structure is well constrained along most of the geotransect, and we slightly modified it when strictly necessary to match the surface observables after trying changes in the geometry and composition of the less constrained mantle bodies. Nevertheless, modifications of the crustal model are always within the uncertainties of the experimental data. The boundaries of the subcrustal bodies must be considered as transition zones where the properties of the mantle (composition, seismic velocities, and temperatures) change gradually rather than abruptly.

The obtained results are subjected to possible variations in the chosen material parameters but the integration of different observables reduces considerably the uncertainty in unraveling the crust and upper mantle structure along the studied profile. Although we are no including a general sensitivity analysis, the reader is referred to previous works in which the same methodology has been used (e.g., Carballo, Fernàndez, Torne, et al., 2015; Tunini et al., 2016).

### 4.1 Crustal and Lithosphere Structure

**Figure 4:**
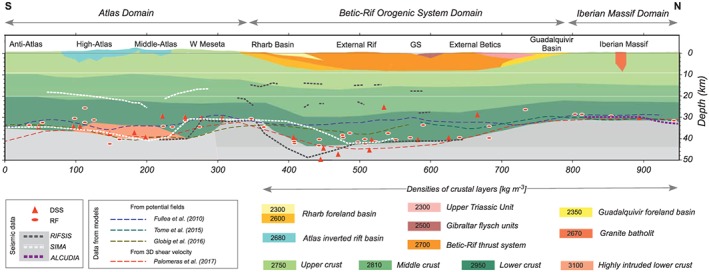
Crustal structure resulting from our modeling, colors are associated to the assumed densities. Different symbols and dashed lines show the Moho depth and internal crustal boundaries from previous studies. RIFSIS (Gil et al., 2014), SIMA (Ayarza et al., 2014), ALCUDIA (Ehsan et al., 2015), and DSS and RF (deep seismic sounding and receiver functions compiled by Diaz et al., 2016).

Figure 4 shows the structure of the crust corresponding to the best fit model. The Moho depth beneath the Iberian Massif is 32 km, increasing toward the Guadalquivir Basin and reaching 40 and 43 km beneath the Betics and the Rif mountains, respectively. Beneath the Rharb Basin the crust shows the shallowest Moho (30 km) increasing again toward the Western Meseta and the Atlas Mountains where it reaches 40 km. In the Atlas region we have considered a 6‐ to 8‐km‐thick high‐density layer (3,100 kg/m^3^) in the lowermost crust related to a pervasive intrusion of mafic rocks linked to the Mesozoic rifting and long‐lasting volcanism.

**Figure 5:**
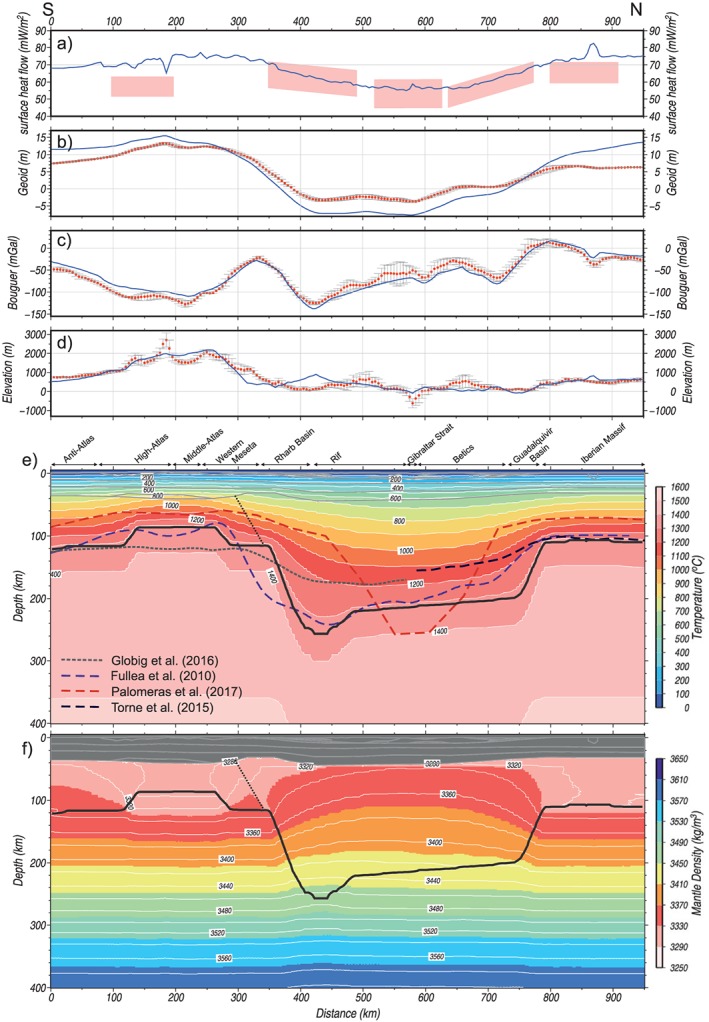
Model results without sublithospheric anomalies. (a) Surface heat flow; pink boxes are mean heat flow values from different authors (see text). (b) Geoid height. (c) Bouguer gravity anomaly. (d) Elevation under local isostasy. Blue lines represent the calculated values from the model. Red dots denote measured data, and vertical bars denote the standard deviation calculated on a strip of 50‐km width. (e) Temperature distribution (color pattern), lithosphere‐asthenosphere boundary (thick black line), and transition between the two lithospheric mantle compositions (thin black dashed line) resulting from our model. Color dashed lines are the lithosphere‐asthenosphere boundary resulting from previous studies. (f) Density distribution.

We have deemed two main lithospheric mantle types according to the geological domains and xenolith samples (Figures 5e and 5f and Table 2). For the Iberian‐Rif domain we use the chemical composition derived from the Calatrava Volcanic Province xenoliths sampled in Cerro Pelado (Sample 65290 in Villaseca et al., 2010) characterized by volatile rich phases indicating metasomatism and secondary magma. For the Atlas domain we adopted the chemical composition of sample Taf19 (El Messbahi et al., 2015) corresponding to the North Middle Atlas with a fertile composition suggesting refertilization. The composition of the sublithospheric mantle is assumed to correspond to a primitive upper mantle following McDonough and Sun (1995). The boundary between these two domains of the lithospheric mantle would roughly correspond to the southern limit of the Rharb basin. This limit has been interpreted subvertical in depth roughly corresponding to the abrupt change of thickness calculated for the upper mantle. Its surface projection could correspond to the continuation of the large fault zone of Nekor, characterized by large outcrops of serpentinites (Chalouan et al., 2008; Michard et al., 1992).

**Table 2:** Major Elements Composition in the NCFMAS System for the Lithospheric Mantle and Asthenosphere Domains Used in the Modeling

Mantle	Atlas	Iberia‐Rif	PUM (primitive upper mantle)
Reference	El Messbahi et al. (2015)	Villaseca et al. (2010)	(McDonough & Sun, 1995)
SiO_2_	45.23	44.51	45
Al_2_O_3_	3.77	3.76	4.5
FeO	9.30	8.75	8.1
MgO	36.46	37.89	37.8
CaO	4.43	3.28	3.6
Na_2_O	0.26	0.36	0.36
Mg#	88.6	0.91	89.3

**Figure 6:**
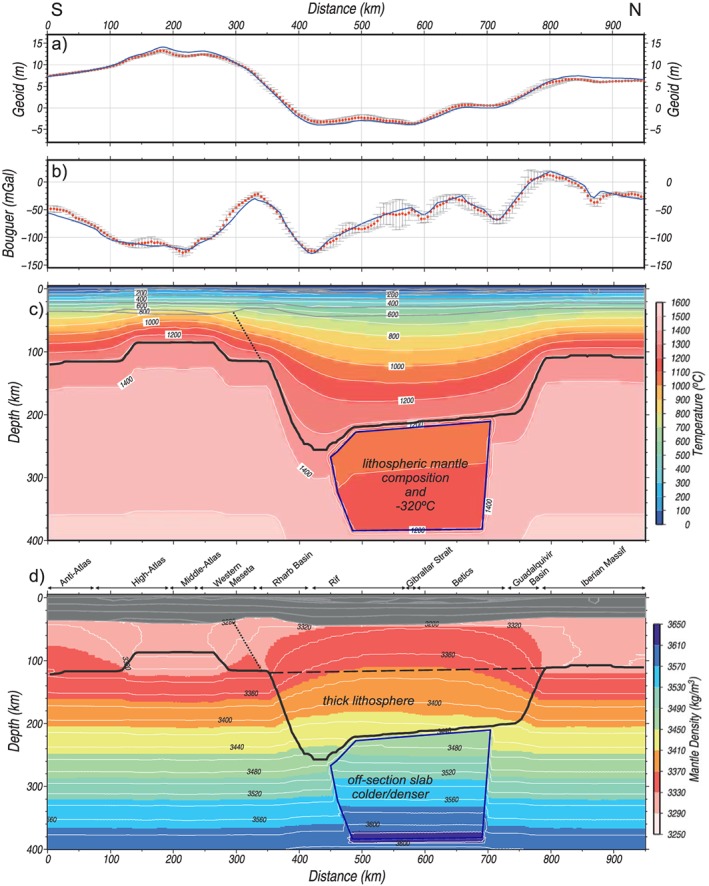
Model results considering a cold‐lithosphere composition at sublithospheric levels. (a) Geoid. (b) Bouguer anomaly. Blue lines represent the calculated values from the model. Red dots denote measured data, and vertical bars denote the standard deviation calculated on a strip of 50‐km width. (c) Temperature distribution. (d) Density distribution. The lithosphere beneath the black dashed line corresponds to the LAB thickening of the Betic‐Rif Orogenic System. The resulting lithosphere‐asthenosphere boundary is shown by a thick black line, and the transition between the two lithospheric mantle compositions by a thin black dashed line. The off‐section cold lithospheric mantle anomalous body (−320°C) is located within the blue line.

Figure 5 shows the best fit model. The calculated heat flow and elevation match the major observed trends along the profile (Figures 5a and 5d), but geoid and Bouguer anomaly show large misfits of long wavelength (Figures 5b and 5c) indicating a mass deficit in the central part of the geotransect. A major caveat is that the modeled geotransect runs across strongly three‐dimensional structures at shallow and deep levels. Tomography models reveal that the lithospheric slab beneath the Betic‐Rif orogen dips radially toward the Alboran Basin, and therefore, the central segment of our geotransect crosses only the upper part of the slab, whereas the deeper part is east off profile, as illustrated in Figure 3 and been reported by previous studies (e.g., Gutscher et al., 2002; Thurner et al., 2014). Despite this, the off‐profile portion of the cold and dense slab will be reflected in the measured potential fields. Hence, we calculate its contribution on the geoid height and Bouguer anomaly by considering a sublithospheric body beneath the External Betics and Rif with a temperature anomaly of −320°C (relative to the surrounding sublithospheric mantle temperature, Figure 6c) and chemical composition corresponding to the Iberian‐Rif lithospheric mantle (Figure 6 and Table 2). The calculated geoid height and Bouguer anomaly fit now much better the observed values, with less than 1‐m error for the geoid and few mGal for the Bouguer anomaly (Figure 6). Note that the results shown in Figure 6 are only significant for the recalculated potential fields but are meaningless in the calculated elevation, surface heat flow, and seismic velocity (*V*_*p*_ and *V*_*s*_). The temperature and density distributions within the mantle are described in the next subsection.

The LAB depth (Figure 5e) is ~110 km beneath the stable Iberian Massif showing a sharp increase up to 200‐km depth beneath the Guadalquivir Basin. From there, the LAB gently deepens to 220 km beneath the Betics and northern Rif, reaching a maximum depth of 260 km beneath southern Rif. Farther south, the LAB rises very steeply to depths of ~120 km below the Rharb Basin and the Western Meseta, with a minimum depth of ~85 km underneath the Middle and High Atlas. The Anti‐Atlas is characterized by a LAB depth of 120 km.

**Figure 7:**
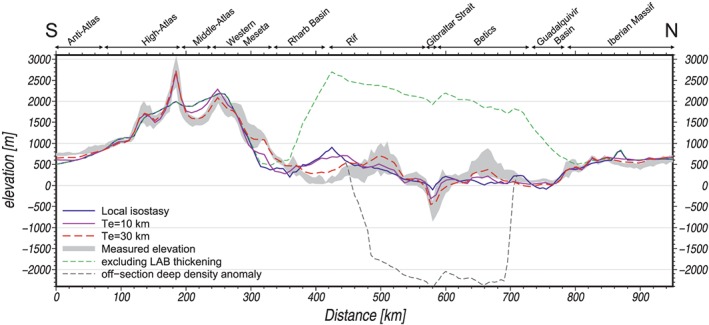
Observed and modeled elevation along the S‐N cross section. Gray: standard deviation of the observed elevation across a 50‐km‐wide strip. Blue: local isostatic model ignoring the off‐section sublithospheric denser body from Figure 6. Purple: regional isostasy with an elastic thickness of 10 km. Red dashed line: regional isostasy with an elastic thickness of 30 km. Green dashed line: elevation predicted without the lithospheric thickening (dashed black line from Figure 6d) beneath the Betic‐Rif orogenic System. Black dashed line: elevation predicted when considering the weight of the off‐section sublithospheric denser body attached to the lithosphere.

The calculated elevation shows short‐wavelength (<100 km) mismatches of ~800 m in the region between the northern part of the Western Meseta and the southern Rif, and to a lesser extent (<500 m) in the External Betics (Figure 5d). However, when the flexural rigidity of the lithosphere is considered and vertical loads associated with the topography misfits are applied, the calculated elevation fits well with observations (Figure 7). An equivalent elastic thickness of *Te* = 10 km is enough to adjust the topography over most of the profile, though higher values of *Te* ≈ 30 km are necessary to fit the elevation between the Atlas and the Rif Mountains within less than 200 m. It is also worth noting that the predicted elevation would be between 1,500 and 2,000 m higher in the Betic‐Rif orogenic system domain without the lithospheric thickening, and the off‐profile sublithospheric low‐temperature anomaly would pull down the elevation ~2,000 m (see section 5).

### 4.2 Temperature and Density Distribution

The temperature distribution in the entire model domain is displayed in Figure 5e. The Iberian Massif is characterized by flat isotherms with a Moho temperature of ~650 °C, whereas in the Betic‐Rif orogenic system the isotherms show a downward deflection related to the slab, with a Moho temperature around 600 °C increasing locally to ~700 °C beneath the southern External Rif. Maximum Moho temperatures are reached beneath the Middle and High Atlas with values between 850 and 900 °C and associated with the upward deflection of the isotherms due to the imposed lithospheric thinning. The lateral temperature variations below the LAB in Figure 5e are due to the lithosphere thickness variations and the restrictions to keep the adiabatic thermal gradient within the asthenosphere.

The calculated mantle density distribution is shown in Figure 5f. The density variations along the profile are mainly due to the different compositions and P‐T conditions. In the Iberian Massif, the lithospheric mantle density increases with depth from 3,280 kg/m^3^ beneath the Moho to 3,315 kg/m^3^ at the LAB. Lithospheric mantle density increases laterally toward the central segment of the profile (Betic‐Rif orogenic system domain) with values ranging from 3,330 kg/m^3^ beneath Moho, to 3,420–3,430 kg/m^3^ at the LAB. Highest lithospheric mantle densities of ~3,480 kg/m^3^ are calculated at the deepest LAB (260 km) beneath the southern External Rif. Note that at the same P‐T conditions (at 300–350 km along the profile), the lithosphere mantle composition of the Atlas domain results in a density that exceeds that from the Iberian‐Rif domain by ~10 kg/m^3^, in average. Therefore, the lowest density values of the lithospheric mantle beneath the Atlas Mountains are due to the thin lithosphere in this region, which becomes in high temperatures at relatively low pressures. Densities in the lithospheric mantle of the Atlas Domain vary slightly, from 3,295 kg/m^3^ below Moho to 3,300 kg/m^3^ at LAB.

At sublithospheric levels, density variations are negligible, reaching maximum values of 3,600 kg/m^3^ at 400‐km depth. Some temperature effects are also seen mainly related to lithospheric thicknesses variations (Figure 5f). The anomalous temperature of −320 °C of the off‐section sublithospheric body representing the nearby cold Betic‐Rif lithospheric slab (Figure 6c) produces a density increase of ~25 kg/m^3^ (Figure 6d).

### 4.3 Upper Mantle Velocity Model

**Figure 8:**
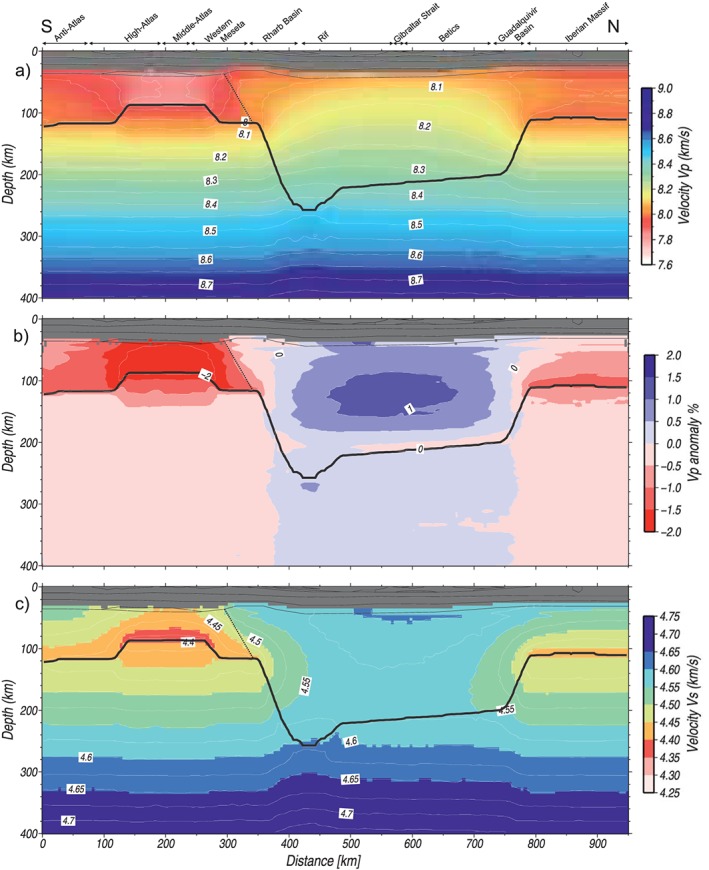
Model seismic velocities results along the profile. (a) *P* wave velocities, (b) *P* wave anomalies with respect to a reference column at 375 km, and (c) *V*_*s*_ velocities.

Our seismic velocity calculations include anelastic effects following (Jackson et al., 2010) and considering a grain size of 5 mm and an oscillation period of 75 s. Figure 8a shows the calculated *P* wave velocities in the mantle. The most outstanding feature is the large lateral velocity variations within the lithospheric mantle mainly related to the variations of the LAB depth along the geotransect. The lithospheric mantle velocity in the Iberian Massif ranges from 7.95 to 8.05 km/s, with the minimum values just beneath the Moho and at the base of the lithosphere. The *P* wave velocities in the Betic‐Rif orogenic system increase from ~8.05 km/s in the uppermost mantle to 8.3–8.45 km/s at LAB depths.

In contrast to density, at the same P‐T conditions, the considered lithosphere mantle composition of the Atlas domain results in lower seismic velocities than those from the Iberian‐Rif domain. This, together with the shallow LAB and hot lithosphere beneath the Atlas Mountains, makes the *P* wave velocity of the lithospheric mantle to range between 7.8 and 7.9 km/s. At sublithospheric mantle levels, *V*_*p*_ increases mostly linearly with depth up to 8.75 km/s at 400‐km depth. Note that seismic velocities are calculated along the profile and therefore, the off‐section sublithospheric anomaly is not imaged. The off‐profile thermocompositional sublithospheric body would increase the *P* wave velocities by ~0.1 km/s.

To compare the computed mantle velocities with global *P* wave models, we calculate the lateral variations of *V*_*p*_ in percent relative to a reference column of the transect located, in our case, at 375‐km distance (Figure 8b). The calculated synthetic tomography reproduces the main trends of slow and fast velocity regions observed in the global *P* wave tomography models (Figure 3c) with minor discrepancies in the amplitudes.

Calculated *S* and *P* wave velocities show a similar pattern (Figure 8c). Minimum values of *V*_*s*_ (<4.4 km/s) are obtained at the base of the lithosphere and the uppermost sublithospheric mantle beneath the Atlas Mountains, whereas *V*_*s*_ in the Iberian Massif range between 4.4 and 4.5 km/s and beneath the Betic‐Rif orogenic system is ~4.6 km/s. Maximum *V*_*s*_ values of ~4.75 km/s are obtained at the base of the model. The calculated *V*_*s*_ pattern compares well with the regional *S* wave tomography model projected onto the transect (Figure 3e) obtained by Palomeras et al. (2017) from ambient noise and teleseismic arrivals. The off‐profile thermocompositional sublithospheric body would increase the *S* wave velocities by ~0.1 km/s.

## 5 Discussion

### 5.1 The Moho and LAB Topography

Since the initial crustal model was constructed using the available seismic and geological data, we will focus on those parts of the transect that were initially not well constrained. The section affecting the Guadalquivir Basin and the External Rif was not imaged by recent DSS experiments and main constraints come from old DSS data, RF analyses, and surface wave tomography. The Moho depth data compiled by Diaz et al. (2016) show large discrepancies, with values of 25–27 km in the southern border of the Guadalquivir Basin and 30–40 km beneath the External Betics. Our crustal model suggests a gentle crustal thickening from the Iberian Massif (30–32 km) to the External Betics and the Gibraltar Strait (~40 km) in very good agreement with Palomeras et al. (2017) (Figure 4).

The section between the Rif Mountains and the Western Meseta also shows noticeable discrepancies in the estimated crustal thickness from the RIFSIS and SIMA deep seismic experiments (Figure 4). The RIFSIS survey (Gil et al., 2014), designed to image the crustal structure beneath the Rif Mountains, shows a maximum crustal thickness of ~48 km, in contrast to the 30–35 km inferred from the SIMA experiment, whose main focus was the Atlas Mountains (Ayarza et al., 2014). Our results show a maximum crustal thickness of ~43 km, in agreement with RF and old DSS data (Diaz et al., 2016) and surface wave tomography (Palomeras et al., 2017), although the style of the crustal geometry looks more like that of RIFSIS experiment. The locus of the crustal thinning from the Middle Atlas to the Western Meseta and the Rharb Basin also differs between both seismic experiments, though the crustal thickness values proposed beneath these structures are coincident. Our results show also similar crustal thickness values but with a noticeable northward displacement of the crustal thinning, which we propose to be located in the Western Meseta, about 50 km more to the north than that proposed by Ayarza et al. (2014). Finally, beneath the Atlas Mountains we have considered a high‐density lower crustal layer of 6‐ to 8‐km thickness to simultaneously fit the potential field data and the low uppermost mantle velocities, which demand high temperatures and therefore a noticeable lithospheric thinning. The thin lithospheric mantle and the concurrence of calc‐alkaline and alkaline volcanism, together with previous models of the region, give support to the hypothesis of crust/mantle melting intruding the lower crust levels.

Figure 5e compares our resulting LAB topography with previous studies by Fullea et al. (2010), Torne et al. (2015), Globig et al. (2016), and the seismic lithosphere by Palomeras et al. (2017). There is a good coincidence between the main LAB trends derived from our study and the 3‐D geophysical‐petrological model by Fullea et al. (2010) all along the transect; as well as, with the models by Torne et al. (2015) along the northern segment and Globig et al. (2016) along the southern segment. Major discrepancies concern to the central segment of the transect where, despite the lithospheric thickening reported by the two aforementioned works beneath the Betic‐Rif domain, the derived LAB depth is 40–70 km shallower. These differences are related to the limitations of the methodology used in these works to calculate the crustal and lithospheric thickness in complex regions as the Gibraltar Arc, characterized by the presence of thick allochthonous low‐density sequences forming the Betic‐Rif orogen, and the subducted lithosphere mantle. The significant lithospheric thinning beneath the Atlas Mountains have been also reported by previous integrated geophysical models with LAB depth values of 80–100 km (e.g., Jiménez‐Munt et al., 2011; Teixell et al., 2005; Zeyen et al., 2005) and interpreted as the consequence of shallow mantle plume (Missenard et al., 2006), edge‐driven convection (Kaislaniemi & van Hunen, 2014; Missenard & Cadoux, 2012), and mantle dripping and lateral dragging (Zlotnik et al., 2014).

The LAB geometry inferred from Rayleigh wave tomography by Palomeras et al. (2017) shows a similar pattern than all the previous mentioned works though LAB depths are ~40 km shallower beneath the northern and southern segments. In the central segment of the geotransect the seismic LAB reaches depths of 250 km, coinciding with the bottom of the tomography model, and shows a narrower shape than our model (Figure 5e). The LAB defined by Palomeras et al. (2017) is the depth of the strongest negative *S*_*v*_ wave velocity (*V*_sv_) gradient once the variation of *V*_sv_ with depth is obtained by inversion of the dispersion curves of the Rayleigh wave phase velocities (see also Priestley & Tilmann, 2009). The surface wave analysis is not very sensitive to sharp velocity changes with depth and therefore, this *S*_*v*_ wave‐derived LAB is a proxy of the base of high‐velocity mantle lid. Although the precise determination of the LAB depth depends on how it is measured (Eaton et al., 2009), the different definitions should show a similar trend as all they are imaging the rheologically strong outer layer of the Earth. In our case, along the northern and southern segments of the geotransect, the *S*_*v*_ wave LAB roughly follows the 1000 ± 50 °C isotherm, whereas in the central segment it largely departs from this isotherm showing higher temperatures. This departure is related to the presence of the subducted cold slab, which is not in thermal equilibrium and has probably average temperatures ~200 °C lower than that calculated in our model.

### 5.2 Mantle Composition

The forward modeling scheme used in our approach forced us, after several attempts, to use different compositions for the lithospheric mantle in the Iberia‐Rif and Atlas domains. A unique composition for the whole geotransect results in large long‐wavelength misfits in the Bouguer anomaly and the geoid height and also in the uppermost mantle seismic velocities, such that fitting the observations requires a denser and seismically slower mantle beneath the Atlas domain relative to the Iberia‐Rif domain.

There is a general agreement that the lithospheric mantle is far to be chemically homogeneous and that it can show a large variability as inferred from geochemical analyses made on numerous xenolith suites. This poses severe difficulties in choosing an appropriate chemical composition that is representative for a whole lithospheric mantel domain. Indeed, geochemical analyses carried out on xenolith suites sampled in the Calatrava Volcanic Province (CVP) and the Middle Atlas reveal important heterogeneities in composition and processes affecting the lithospheric mantle.

The CVP xenoliths are affected by two metasomatic events indicating mixing between asthenospheric mantle and deeply recycled enriched mantle from oceanic subduction (e.g., Bianchini et al., 2010; Villaseca et al., 2010). Using the chemical composition derived from the CVP xenoliths to the whole Iberian‐Rif domain is justified by the spatial continuity of the low seismic velocities along a narrow “channel” extending WSW from the CVP to the Gulf of Cadiz and surrounding the Betic‐Rif orogen. This low velocity channel is imaged by multiple‐frequency *P* wave tomography models at depths of 60–200 km (Figure 3; Bezada et al., 2014; Bonnin et al., 2014) and surface wave tomography models at depth of 75–125 km (Palomeras et al., 2017).

In the Middle Atlas the xenolith samples analyzed by El Messbahi et al. (2015) represent a lithospheric mantle section variably depleted by extraction of basic melts and show a clear HIMU (high U/Pb ratio) signature. In particular, the samples of Tafraoute maars in the North Middle‐Atlas, dominated by fertile spinel lherzolites, record heterogeneous infiltrations of small sublithospheric melt fractions preserving the signature of extensional lithospheric thinning during Mesozoic. The abundance of extensively refertilized samples and Fe‐rich wehrlites suggests that wide sectors of the mantle beneath the NE‐SW volcanic alignment consist of rejuvenated lithosphere.

For consistency with previous works, in the Iberia‐Rif domain we have considered the same mantle chemical composition as Carballo, Fernàndez, Torne, et al. (2015) used for the CVP, which corresponds to a moderately depleted lithosphere mantle relative to the primitive upper mantle due to a very low partial melting event (1.5%) (Table 2). The need of increasing the average mantle density and decreasing the seismic velocities in the Atlas domain relative to the Iberia‐Rif domain to fit the geophysical data can be accomplished with a composition richer in FeO and keeping the Al_2_O_3_ content (Tunini et al., 2016). Such is the case for the chosen mantle xenolith composition in the Middle Atlas, which at given P‐T conditions results in higher density (~7 kg/m^3^, 0.21%) and lower seismic velocities (~0.035 km/s, 0.44%) than the Iberian‐Rif domain composition. Compositions for the Atlas domain enriched in FeO and depleted in Al_2_O_3_ relative to the Iberia‐Rif domain, as is the case of some sampled xenoliths (El Messbahi et al., 2015), could also match the seismic velocities and density‐dependent observations. It is worth noting that the variations on density and seismic velocities in the mantle due to pressure and temperature changes are higher than due to chemical composition changes. Assuming the same composition, a temperature increase of 200 °C at 75‐km depth will decrease the density by 32 kg/m^3^ and the *P* wave velocity by 0.113 km/s, while increasing the depth by 20 km, will increase the density by about 23 kg/m^3^ and the *P* wave velocity by 0.08 km/s. At this point, it must be kept in mind that different mantle compositions can result in the same bulk density and seismic velocities, and therefore, there is a nonunique solution concerning to the chemical composition of the lithospheric mantle (Afonso et al., 2013).

### 5.3 Thermal and Compositional Sublithospheric Anomaly

**Figure 9:**
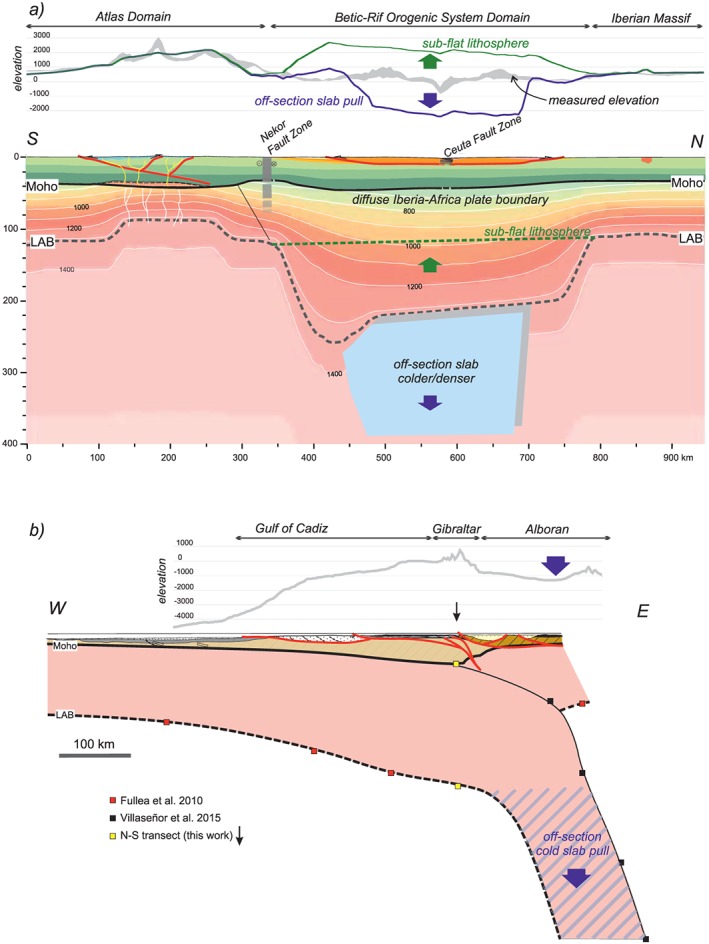
Two lithospheric profiles crossing into Gibraltar, localization in Figure 2a. (a) S‐N profile, resulting crustal structure and temperature distribution from this work. The top panel shows the measured elevation (gray), the uplifted elevation considering a flat LAB beneath the Betic orogenic System (green), and the pulled down elevation considering the off‐section slab (blue). (b) E‐W cross section, perpendicular to our profile. Crustal structure from Iribarren et al. (2007) and LAB and slab top from previous works (Fullea et al., 2010; Villaseñor et al., 2015). The slab beneath the Alboran Sea corresponds to the “off‐section slab” affecting the geoid and gravity calculations along our profile, and it is pulling down the Alboran basin.

Our results (Figure 5) show that fitting the observed gravity and geoid data requires the influence of the cold and denser off‐profile lithospheric slab, which is incorporated to the model as a thermal and compositional sublithospheric anomaly (Figure 6). If the negative buoyancy of this slab were entirely transmitted onto the profile, it would produce a negative topography of ~2,000 m (Figures 7 and 9a). However, the slab is attached to the Iberia‐Rif lithosphere some tens of kilometers to the east of the Gibraltar Strait (Figure 3d), where bathymetry increases up to 1,400 m toward the Western Alboran Basin and the basement lies at depths exceeding 9 km (Figure 9b; Torne et al., 2000). Therefore, though the thermocompositional sublithospheric anomaly simulating the subducting slab has noticeable effects on the gravity and geoid fields, the slab‐pull force and its effects on topography is mainly noticed in the back‐arc Alboran Basin and are negligible along the profile.

### 5.4 Tectonic Relevance

The present‐day structure of the crust and lithosphere mantle in the study region is the result of a complex tectonic evolution that initiated with the formation of the Pangea supercontinent. Both Iberia and Africa belonged to the Gondwana continental assemblage that collided with Laurentia building the Variscan orogen during the Late Paleozoic. The collapse of the Variscan orogenic system occurred by extensive and long‐lasting lithospheric rifting during Permian and Triassic times culminating with the emplacement of the massive Central Atlantic Magmatic Province in the Triassic‐Jurassic boundary (Marzoli et al., 2018). The Early Jurassic to present spreading of the Central and Northern Atlantic Ocean constrained the tectonic motions among African, Iberian, and European plates characterized by a broad transtensional regime creating the highly segmented Ligurian‐Tethys Ocean between Africa‐Iberia and Iberia‐Adria. Significant extension occurred within the continental blocks, the most prominent being the Atlas basin in NW Africa and the Iberian basin in the Iberian microplate. Most of these Jurassic‐Early Cretaceous basins overprinted previous Permo‐Triassic thinned crustal zones. During latest Cretaceous time, the northern convergence of Africa against Europe deformed both microplate boundaries (Pyrenees continental collision to the north and Betic‐Rif subduction‐related orogen to the south) as well as the interior of the Iberian microplate (Iberian Ranges, Catalan Coastal Ranges, and Central System) (Vergés et al., 2019).

The imprints of this tectonic evolution are evident in the resulting crust and upper mantle structure. At crustal levels, we have considered a layered crust consisting of upper, middle, and lower crust with homogeneous densities all along the profile and varying their respective thicknesses, which result in lateral crustal density variations that can be attributed to major lithological changes. It must be noted that our modeling approach is particularly sensitive to the density distribution, and therefore, the geometry of the crustal layers responds to the density variations required to fit the observations (gravity, geoid, and elevation) rather than to geological structures resulting from tectonic deformation.

The average density of the crystalline crust in the outcropping Iberian Massif (Centro‐Iberian and Ossa‐Morena zones) is of ~2,820 kg/m^3^ increasing toward the Betic‐Rif orogenic system to values of ~2,850 kg/m^3^, in agreement with the lithological changes across the Iberian Massif domains. This density increases in the Ossa‐Morena and SPZs has also been reported by Torne et al. (2015) from a 3‐D gravity analysis over the whole Iberian Peninsula. The Anti‐Atlas domain shows average densities similar to that of the crust underlying the Betic‐Rif orogen (~2,850 kg/m^3^) indicating a predominant metamorphic and igneous rock composition. These values increase to 2,880–2,885 kg/m^3^ beneath the Atlas Mountains due to the mafic high‐density lowermost crustal layer.

From the interpretation of the SIMA deep seismic experiment in the Atlas, Ayarza et al. (2014) propose a crustal‐scale NW dipping thrust offsetting the Moho under the northern part of the Middle Atlas. This interpretation agrees with the decrease in the average crustal density toward the Western Moroccan Meseta.

The subparallel trace of the geotransect to the frontal structures of Betic and Rif fold‐and‐thrust systems only allows a limited inspection of their tectonic structures. The allochthonous units of the Betic and Rif fold belts, including the Gibraltar Flysch unit and the frontal Triassic diapiric unit, thrust over the Guadalquivir Basin to the NW and the Rharb Basin to the SW. The Betic fold belt deforms the SE Iberian Ligurian‐Tethys margin and the Rif fold belt deforms the NW Moroccan Ligurian‐Tethys margin. Basement beneath the Guadalquivir and Rharb basins gently dip beneath the basal thrusts of the Betic and Rif thrust systems, respectively. Because there are no gravimetric anomalies under the Betic‐Rif thrust systems along the study geotransect, it is interpreted that the Internal Zones of both systems are not cut by the profile or alternatively that their volume is not enough to cause perturbations.

The presence of a high‐density lower crustal body beneath the High and Middle Atlas is explained by the Triassic‐Jurassic extension that necessarily produced crustal thinning to accommodate several kilometers thick Jurassic sedimentary sequences and long‐lasting volcanism with mantle affinity. The present crustal thickness in the Atlas region would be the result of pervasive intrusion of magma in the lower crust and of tectonic shortening associated with the Africa‐Eurasia convergence. It is interesting to note that the growth of many of the anticlines ridges of the Central High Atlas during the Jurassic Period might reduce the amount of calculated Cenozoic shortening for the Atlas Mountain chain as discussed by Moragas et al. (2017).

The gravity and geoid measured along the transect are consistent with the presence of a large body deeper than 200 km underneath the Strait of Gibraltar and about 25 kg/m^3^ denser than the surrounding mantle (Figure 6). This density contrast is consistent with a temperature anomaly of −320 °C and the chemical composition from the Iberian‐Rif lithospheric mantle. However, a deeper part of this high‐density lithospheric slab does not have an isostatic impact on elevation and is barely visible in the seismic *P* wave velocity section (Figure 3c). Seismic tomography imaging (e.g., Bezada et al., 2013; Bonnin et al., 2014; Civiero et al., 2018; Villaseñor et al., 2015) shows that, at 200‐km depth, this anomaly is far enough from the Strait to have an isostatic effect while still affecting the potential fields, as shown from flexural models of the Guadalquivir Basin (Garcia‐Castellanos et al., 2002).

The calculated elevation would be about 1,500–2,000 m higher in the central area of the section (Betic‐Rif orogen) without the lithospheric thickening (long dashed line Figure 6d). This represents an estimation of the amount of slab pull that has sunk the strait of Gibraltar since the Messinian salinity crisis (Garcia‐Castellanos & Villasenor, 2011), purportedly triggering the reflooding of the Mediterranean basin after a kilometric sea level drawdown around 5.5 Ma.

The weight of this slab can also explain the subsidence and tilting of the Guadalquivir basement during and after the upper Miocene (Garcia‐Castellanos et al., 2002; Pérez‐Asensio et al., 2018). In Iberia, regions of crustal thickness of ~40 km correspond to areas higher than 1,000 m, as, for example, in the Pyrenees (Carballo et al., 2015). The lack of such high elevation in the Strait of Gibraltar points to a subcrustal downward force pulling down the area. Our results support the idea that the strait is depressed by the weight of a cold lithospheric slab hanging in the mantle between the Rharb and the Guadalquivir basins.

## 6 Concluding Remarks

The presented geophysical‐petrological model along the N‐S trending geotransect, from the Iberian Massif to the Anti‐Atlas in NW Africa, through the Gibraltar Arc shows the crustal and lithospheric structure in a complex tectonic region where there are large and recently acquired geophysical databases (DSS, RF analyses, and *P* and *S* wave tomography).

The crust, constrained by seismic results, is thicker (deeper Moho) beneath the orogenic systems of Betics and Rif and under the Atlas Mountains, reaching about 40, 43, and 40 km, respectively. The Betics and Rif orogens are depicted as thin skinned along the geotransect whereas the Atlas Mountains are interpreted as thick skinned by the product of basin inversion during the Cenozoic. The crust underneath the Hercynian Iberian Massif is about 32 km thick, whereas the crust beneath the Anti‐Atlas is slightly thicker reaching 35 km of thickness. The thinner crustal domain corresponds to the segment under the northern Western Meseta with about 30 km, possibly corresponding to the NW African margin during the Jurassic.

The LAB, however, shows significant lateral variations in thickness (110–260 km) and composition, showing a good match with their respective crustal domains characterized by different tectonic evolutions. Our results compare well with the general trends of previous models and show a better resolution and slightly deeper values. From north to south, the Hercynian Iberian Massif shows a steady LAB located at 110‐km depth. An abrupt increase of this depth is noticeable toward the Betics and Rif tectonic domains with values at 220 and 260 km, respectively. We link this lithospheric thickening to the lithospheric slab visible in seismic tomography and estimate that it may have pulled down the topography of the Strait of Gibraltar by about 1,500–2,000 m. The latest part of this subsidence could be responsible for the reconnection of the Atlantic Ocean and the Mediterranean Sea, leading to the reflooding of the Mediterranean after the Messinian salinity crisis.

This maximum depth of the LAB under the Rif is limited by the Gibraltar Strait (Ceuta Shear Zone) with small lenses of peridotites and by the Nekor Fault Zone along which large serpentinite units crop out. We interpret the Gibraltar‐Ceuta and the Nekor broad fault zones as expressions of deep faults, active during the sinistral drift of Africa with respect to Iberia during the Jurassic period, separating the Iberian microplate from the NW Moroccan block.

To the south of the Nekor Fault, the lithospheric mantle is modeled denser and seismically slower for its correct adjustment. The chemical compositions of the Atlas and Betics lithospheres are compatible with xenolith data sampled in the Middle Atlas and the Calatrava Volcanic Province, respectively. This change in the upper mantle reinforces the idea of a boundary between the African mantle and the Ligurian Tethys one.

The geometry of the mantle is intriguing, however, below the Atlas Mountains, characterized by a thick crust, under which the LAB is located only 90 km deep. This thinning of the LAB, regardless the Cenozoic shortening, long lasting as indicated by Triassic‐Jurassic to Present volcanism, is related to deeper geodynamic processes. The LAB under the Anti‐Atlas shows a relatively flat surface at 120 km and therefore very similar to that of the Iberian Massif to the north.

The Ligurian‐Tethys subducting‐delaminated slab beneath the Betic‐Rif orogenic system is incorporated into the model as an off‐profile thermocompositional sublithospheric body with a temperature anomaly of −320 °C and chemical composition as the Iberian‐Rif domain.

The obtained lithospheric structure across the Iberia‐Africa boundary is compatible with a subduction‐related orogenic system related to the consumption of the Tethys lithosphere that produced a significant northwest and west directed rollback. During rollback the cover units of both Iberian margin (Betics) and NW African margin (Rif) were imbricated ahead of the tectonically emplaced Internal metamorphic units.
